# Do taxpayers consider quality labels as a form of recommendation for good tax-preparation services? Evidence from Germany

**DOI:** 10.1016/j.heliyon.2023.e23357

**Published:** 2023-12-19

**Authors:** Johannes Wichmann, Michael Leyer, Isabell Altmüller

**Affiliations:** a Philipps-University of Marburg Barfüßertor 2, 35037 Marburg, Germany; b Queensland University of Technology 2 George St, Brisbane, QLD 4000, Australia; c KPMG Germany, Fuhlenwiete 5, 20355 Hamburg, Germany

**Keywords:** Reasoned action approach, Principal–agent theory, Tax-preparation services, Accounting services, Taxpayer, Quality label

## Abstract

Quality labels are important measures for reducing the information asymmetry between principals and agents to enhance trust. However, little research is available on the effects of quality labels used in tax-preparation services from critical perspectives in accounting. The present research contributes to the field by addressing the underlying reasons for German taxpayers to consider quality labels for identifying good tax-preparation services. These contributions are based on a survey of 199 taxpayers in Germany. The results show that the intention to consider quality labels is mainly driven by taxpayers' attitude and trust in quality labels, perceived norms having some influence, and perceived behavioral control having no influence. Hence, we highlight that the reasoned action approach is appropriate for this study, and aim to identify the relevant factors that influence taxpayers’ intention to consider quality labels. The results contribute to a general understanding of the importance of quality labels for tax-preparation services in the context of accounting, organizations, and society. Both academia and practitioners can utilize these insights for further research and consultation on quality labels and tax-preparation services.

## 1 Introduction

Sufficient tax reporting is mandatory for all businesses and individuals [1]. Because tax-related tasks are known to be complex and error-prone [2], businesses and individuals often seek help to accomplish them and report taxes appropriately [3]. Tax preparers are professional service providers [4,5] that help businesses and individuals with tax-related tasks [3,6,7]. However, the relationship therein is characterized by information asymmetry between tax-preparation services on one side and businesses and individuals as taxpayers on the other side [8,9]. Such information asymmetries, as critical perspectives, have been the subject of various studies in accounting (see, e.g., Refs. [3,[8], [9], [10], [11], [12]]). For the analysis of these asymmetries, principal–agent theory [13] is the most prominent and has often been used in accounting [10]. Several measures are appropriate for addressing information asymmetries, but quality labels are particularly important in accounting [3,11]. Quality labels are especially relevant to tax-preparation services, as they serve as recommendations and suggest that tax-preparation services are working in the interests of taxpayers and to a high quality [3]. Given that tax-preparation services utilize professional service providers, recommendations are vital for attracting clients [[3], [4], [5],^11^]. Witherspoon and Stone [3] investigated the influence of quality labels on tax-preparation services by analyzing user ratings, while Lee [11] compared quality labels in the UK and US audit professions. However, research in this area overall has been limited. Witherspoon and Stone [3], and Lee [11] stated that quality labels are important for behavioral decisions concerning good tax-preparation services; however, these authors failed to investigate the influencing factors leading to quality labels’ usage. Addressing such a gap is important because quality labels, such as recommendations, have the potential to reduce information asymmetries between taxpayers and tax-preparation services [3,10]. However, no study to date has focused on why quality labels are important. We aim to close this gap, and pose the following research question:

Which factors influence a taxpayer's intention to consider quality labels when choosing good tax-preparation services?

We utilize the reasoned action approach (RAA) as a causal model, as this is widely accepted in psychological studies [14,15]. The approach has previously been applied to accounting [12], and is suitable for determining behavioral intentions. RAA suggests that the intention toward a certain behavior (e.g., considering quality labels to identify effective tax-preparation services) is influenced by behavioral, normative, and control beliefs [16]. We used data gathered from a questionnaire distributed to 199 German taxpayers to identify factors relevant to the intention to consider quality labels and identify effective tax-preparation services. In this scenario, Germany is of special importance with regard to taxpaying, because it boasted the second-highest tax wedge among all organization for economic co-operation and development (OECD) countries in 2022 [17]. This is further supported by the fact that Germany's tax code complexity is far below average among OECD countries [18], indicating an urgent need for taxpayers to consider certain factors when identifying effective tax-preparation services. By using those services and keeping the results of our study in mind, taxpayers can navigate tax code complexity and increase their tax refunds. Structural equation modelling (SEM) shows that RAA is an appropriate approach for research in this context. Furthermore, the findings identify factors that influence the intention to consider quality labels. Therefore, the results contribute to our general understanding of why taxpayers tend to consider quality labels, and their reasons for doing so. To the best of our knowledge, this study is the first to address the psychological reasons for considering quality labels in accounting-related contexts. Thus, this study contributes to accounting research by proposing factors that should be addressed to ensure that tax-preparation services can successfully use quality labels to reduce information asymmetries and, hence, to mitigate the principal–agent dilemma [3,10] between tax-preparation services and taxpayers in general. Tax-preparation services can use these insights to better understand the behavioral drivers of customers. We recommend that tax-preparation services implement measures that qualify them to receive certain quality labels.

The remainder of the article proceeds as follows. Section 2 outlines the theoretical background and presents the hypotheses. Section 3 details the materials and methods. Section 4 presents the results discussed in Section 5. Finally, Section 6 concludes the study and presents an outlook for further studies.

## 2 Theoretical background

### 2.1 Quality labels for tax-preparation services

Quality labels represent specific commitments of certain products and/or processes to adhere to given standards [11]. They are either issued by the company itself or by third parties, such as governments [3] or companies/institutions [19]. Quality labels serve as recommendations and aim to increase the trustworthiness of companies for individuals [3]. In accounting contexts, recommendations (and quality labels) are mandatory for the success of tax-preparation services because research has shown that tax-preparation services who receive more recommendations perform better than their competitors [3]. Specifically, quality labels for tax-preparation services should be designed to enhance trust, since trust is the most important characteristic of tax-preparation services according to taxpayers’ perceptions [9].

### 2.2 Tax-preparation services and taxpayers in Principal–Agent theory

Tax-preparation services utilize professional service providers, and recommendations are mandatory for their success [4,5,7,9,20], as are quality labels [3,11]. According to principal–agent theory, principals (i.e., taxpayers in the context of this study) hire agents (i.e., tax-preparation services) [10,13,21] because they want to complete tasks that they cannot, for many reasons (e.g., limited time, insufficient knowledge, high costs), complete by themselves [6,10,[22], [23], [24], [25], [26]]. Therefore, agents promise that the tasks in question will be completed adequately [13]. Owing to the information advantage that tax-preparation services have over taxpayers, a principal–agent dilemma exists [13]. This dilemma arises because tax-preparation services can exploit their advantage to further their own interests [10]. Sakurai and Braithwaite [9] supported these propositions, as they investigated Australian taxpayers' perceptions of their idealized tax practitioners. By studying the practice of three different types of tax practitioners—(1) creative, aggressive tax planning; (2) cautious minimization of tax; and (3) low risk, no fuss—they determined that most taxpayers prefer low risk, no fuss. Thus, taxpayers prefer to receive a lower tax refund with choosing a tax-preparation services that acts legally compliant instead of aiming for high tax refunds with the risk of not being legally compliant. These propositions were supported by Stephenson et al. [26], who countered the principal–agent dilemma through an exploratory examination of expectation gaps between taxpayers and tax preparers over tax-preparation services. They applied the taxpayer motivation scale, which contains taxpayers' motivations to use tax-preparation services [24]. This scale identifies four reasons taxpayers hire tax-preparation services: (1) to save money, (2) to save time, (3) to ensure legal compliance, and (4) to receive protection from tax authorities. The taxpayer motivation scale is built on Stephenson's [23] proposition, which assesses whether taxpayers align with preparers' self-assessment regarding the extent to which they advocate for their taxpayers. To investigate this phenomenon, the Mason–Levy Advocacy Scale was used, and Stephenson [23] determined that local and regional tax-preparation services tend to be more aggressive in terms of wages, as most taxpayers prefer. Extending this, Stephenson et al. [26] determined that tax-preparation services need to (1) better understand taxpayers' motivations for utilizing them and (2) realign their marketing efforts toward educating taxpayers about the quality of tax services.

Thus, as tax-preparation services improve tax revenue performance for companies [8], and since tax-preparation services need to realign their marketing efforts [26], measures are needed to separate effective from poor tax-preparation services though signals such as quality labels [3].

### 2.3 Related work

Concerning tax-preparation services, Lee [11] investigated quality labels within the auditing profession, and their perceptions among UK and US individuals and companies. Specifically, he was curious about expectation gaps and the role quality labels play in the perception of financial reportings in estimating whether quality labels indeed represent quality in terms of sufficient tax services. He stated that quality labels signal the existence of a formal body of knowledge possessed by auditors. The quality labels indeed provide meaning to the quality of reported tax-related statements [11]. Hence, Lee proposed that quality labels are important in the assessment of auditing professions and should be investigated by future research [11]. In their study investigating determinants of tax-perparer usage examining panel data, Christian et al. [22] provide factors that positively influence individuals’ intention to use tax preparer. They determined three key aspects: first, time-cost-savings for taxpayers are positively influencing the intention to use, with intention increasing for those taxpayers who want to save time. Second, the intention to use is dependent on the type of tax return the individuals are aiming at as well as the respective employment status of the individual. The more complex the type of tax return and the better the employment status of the individual, the more likely the individuals will use a tax-preparation service. Third, the effect of income and marginal tax rate on the intention to use tax-preparation services is unclear for models that aim to explain complexity and when the source of the income of the respective individual is unclear. Nonetheless, Christian et al. [22] state that future research should address certain factors for tax-preparation-service usage continuously, as new legal regulations for taxes could lead to different intentions to use tax preparation services. To report on certain demands for tax preparations, Dubin et al. [6] used nested logit techniques. They distinguished their analysis concerning non-paid preparation-services, paid-third-party preparation-services, and self-preparation of tax services. They determined that education is related to self-preparation, with increases in the percentage of self-preparation for individuals with high school degree or above. Further, they found that taxpayers older than 65 prefer to use tax preparation services that provide them with legal representation towards the United States Internal Revenue Service. Next to age being relevant for the intention to use tax preparation services, they estimated that tax increases lead to a higher intention to use. With using their taxpayer motivation scale and linear regressions, Stephenson et al. [26] ascertained that gender is important for the intention to use tax-preparation services. They found that women are more curious about their tax revenues than men are. Further, they state that taxpayers with children are more curious about saving time and money during their tax-preparing services, compared to those taxpayers without children. Hence, Stephenson et al. [26] support the propositions of time-cost-savings being relevant for the intention to use tax-preparation services stated by Christian et al. [22]. According to Stephenson et al. [26], future studies should also consider the size of the tax-preparation service, as small and regional tax-preparation services tend to be more taxpayer-focused than larger ones in non-regional areas are. On a corporate level, Gleason and Mills [8] determined that auditor-provided tax services improve the estimate of tax revenues. They did, as they used internal revenue service-, financial statement-, and auditor fee data for the estimation in logistic regressions. Their results reflect that investigating tax-preparation services and taxpayers situation using principal-agent theory for explanation is indeed sufficient. They concluded that tax-preparation services must have an information advantage towards taxpayers concerning tax services, as tax revenues were higher for those taxpayers that used tax-preparation services compared to those who did not use them. This perception is also confirmed by Cloyd [2]. He performed a study with 63 tax professionals having to solve a tax-related task and estimated that those tax professionals who had a larger amount of prior knowledge outperformed those with little or no prior knowledge. Hence, according to Gleason and Mills [8] and Cloyd [2], an information asymmetry between taxpayers and tax-preparation services exist and that the better informed the tax professionals are, the larger is the information asymmetry in terms of tax-related services. To gain information about how referrals between professional service providers are perceived, Wheiler [4] used three datasets for his study by investigating referrals for accounting, banking, and law. For accounting, he ascertained that referrals are indeed important for tax-preparation services in order to increase their number of mandates. He did so, as he found out that companies tend to use tax-preparation services when they are referred to or have used the respective tax-preparation service before. In another study addressing quality labels in the context of tax-preparation services, Witherspoon and Stone [3] investigated Yelp ratings of services by separating them into different groups. Via linguistic analyses, the authors found that state-certified tax-preparation services (and thus those perceived as high quality due to having state certification) received better ratings on recommendation portals compared to uncertified ones. Hence, Witherspoon and Stone [3] recommend that tax-preparation services should seek to qualify for state certifications in order to receive better ratings and ultimately obtain more new clients. Further, this proposition is supported by Sakurai and Braithwaite [9] who state that for Australian taxpayers, trust towards tax-preparation services is important for the intention to use. Thus, they display the necessity to not just investigate trust towards tax-preparation services but also to evaluate certain measures to improve trust towards those services. Ultimately, apart from the Witherspoon and Stone [3] study concerning taxpayer ratings, there is a research gap in terms of whether taxpayers consider quality labels as a deciding factor when choosing good tax-preparation services, and the reasons for these considerations. Hence, there is a clear lacuna in our understanding of the reasons why quality labels are (or are not) considered when selecting tax-preparation services.

### 2.4 Reasoned action approach

To ascertain the intention to consider quality labels when identifying effective tax-preparation services, we use a psychological approach, with RAA as the underlying theory. RAA stems from a combination of the widely accepted theory of reasoned action [[27], [28], [29], [30], [31]] and the theory of planned behavior [32] to explain individuals' behavior. According to RAA, behavior is rooted in behavioral intention, which is influenced by three key determinants: (1) the opinion the individual has toward the behavior, defined as the individual's attitude toward the behavior; (2) others' influences that are important to the individual, represented as perceived norms; and (3) the possibility that the individual can actively influence the behavior, called perceived behavioral control [16].

First, attitude describes the positive or negative feelings an individual has toward performing the behavior in question. To determine an individual's attitude, their behavioral beliefs toward the attributes and characteristics associated with the behavior (or the behavior's objective) are assessed. Therefore, overall attitude is influenced by the individual impacts of the behavior and the desirability assessments of those impacts. Second, perceived norms represent the individual's perception of whether other individuals think they should engage in the targeted behavior, calculated as the sum of the perception and motivation assessments related to all relevant referents [[28], [29], [30]]. Normative beliefs moderate these perceived norms, and pertain to relevant reference groups who oppose or support targeted behavior. Third, perceived behavioral control refers to the individual's capacity or control with respect to performing a targeted behavior. This is determined by control beliefs, which represent personal or situational factors that an individual deems important to the targeted behavior. Fourth, intention toward behavior is ascertained by attitudes toward behavior, perceived norms, and perceived behavioral control. Therefore, the more positive these variables are, the more likely it is that the individual will execute the targeted behavior [16]. Ultimately, performance of the targeted behavior entails a process of comparing and selecting among the attitudes, perceived norms, and perceived behavioral controls associated with each of the other attributes in the choice set [33].

### 2.5 Hypotheses and research model

**Fig. 1:**
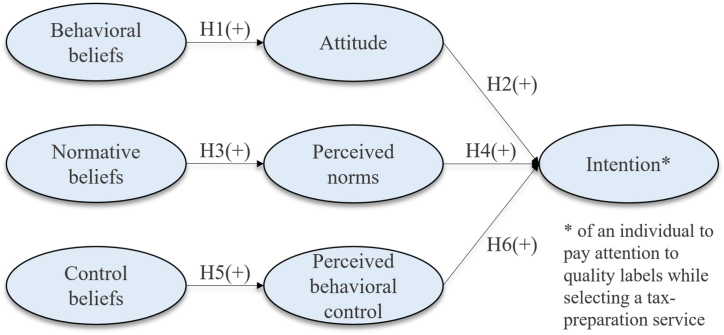
Research model.

As Fishbein and Ajzen [16] stated, RAA must be adapted to the specific context. We do so here by considering quality labels in relation to identifying effective tax-preparation services (for our RAA model, see Fig. 1). First, to ascertain an individual's value perception when considering quality labels to identify effective tax-preparation services, behavioral beliefs must be considered [16]. Concerning quality labels for good tax-preparation services, behavioral beliefs refer to whether individuals think that quality labels are appropriate signals of effective tax-preparation services, which would in turn reduce the principal–agent dilemma [3,10]. These beliefs affect the individual's attitude toward quality labels for effective tax-preparation services. In terms of attitude, we consider whether individuals think that quality labels are appropriate indicators of effective tax-preparation services [3,16,33]. This leads to our first set of hypotheses.

H1 *The higher the behavioral beliefs concerning the consideration of quality labels to identify effective tax-preparation services, the more positive an individual's attitude is toward quality labels for tax-preparation services.*

H2 *The more positive an individual's attitude is toward quality labels in identifying effective tax-preparation services, the higher their intention to consider quality labels when selecting tax-preparation services.*

Second, we identify normative beliefs as others' opinions regarding the behavior in question. In this study, we refer to family, close friends, and individuals who are in a collegial relationship with the individual in question (e.g., corporate colleagues or collegial entrepreneurs). We do so because, according to both RAA [16] and taxpayers' perceptions of idealized tax practitioners [9], friends, family, and colleagues are important reference groups in the service context. The normative beliefs of these groups influence individuals' perceived norms [16]. Regarding perceived norms, we investigate the extent to which an individual values others’ opinions. Thus, in the context of this study, recognizing the value of recommendations from reference groups is necessary when considering quality labels as signifiers of effective tax-preparation services [9,16]. Thus, our second set of hypotheses is as follows.

H3 *The higher the normative beliefs concerning the consideration of quality labels to identify effective tax-preparation services, the more positive an individual's perceived norms regarding quality labels for tax-preparation services.*

H4 *The more positive an individual's perceived norms regarding the consideration of quality labels to identify effective tax-preparation services, the higher their intention to consider quality labels for tax-preparation services.*

Third, control beliefs facilitate or impede behavioral intentions [16]. In this study, control beliefs pertain to whether an individual utilizes quality labels to identify effective tax-preparation services, and whether they are able to assess whether quality labels are helpful in identifying effective tax-preparation services. Hence, our control beliefs investigate whether our individuals have control over certain measures to reduce expectation gaps in providing professional services [7]. These control beliefs then lead to perceived behavioral control, which determines whether the individual is capable of, or has the control for, considering quality labels when choosing good tax-preparation services [3,16]. Hence, the third set of hypotheses is as follows.

H5 *The higher the control beliefs concerning the consideration of quality labels to identify effective tax-preparation services, the more positive the perceived behavioral control of an individual regarding their consideration of quality labels for tax-preparation services.*

H6 *The higher an individual's perceived behavioral control in terms of the consideration of quality labels to identify effective tax-preparation services, the higher their intention to consider quality labels for tax-preparation services.*

Fig. 1 represents the hypotheses (in line with RAA) embedded in the research model.

## 3 Materials and methods

### 3.1 Participants and data collection

In October 2020, we used the crowdworking platform Clickworker (similar to Amazon Mechanical Turk) to recruit German taxpayers. We included test questions at the beginning and end of the questionnaire to ensure that the self-reported status was correct. Furthermore, we obtained informed consent from all participants concerning the processing of the acquired questionnaire data for research purposes. As the unsupervised online platform paid participants for their responses, we followed the recommendations of Goodman et al. [34] by keeping the questionnaire short and enriching it with attention checks. On this platform, the questionnaire appeared as a task that was solvable by anyone who (a) lived in Germany, (b) could answer the survey (since it was conducted in German), and (c) was at least 18 years old. We did not further influence the composition of the participants. As 199 individuals were paid for completing the questionnaire using Clickworker, and could thereby complete the questionnaire quickly, without fully considering the questions, we followed the recommendations of Goodman et al. [34]. They determined that enriching questionnaires with attention-check questions leads to completed questionnaires whose data are similar to those of voluntary participants. Concerning the age of our participants, the youngest was 19 years old and the oldest was 69 years old. The mean age was 39.27 years, with a variance of 135.41 years and a standard derivation of 11.64 years. In addition, 77.39 % of participants were between 18 and 49 years old, and 22.61 % were in the age range of 50 and 69 years. With respect to the year in which participants had first consulted a tax-preparation service, 85 had never consulted one before, 16 had consulted one before 2016, and 98 had consulted one after 2016. Additionally, we investigated the most important task for which an individual would utilize a tax-preparation service. The answers included (1) preparation of annual income tax return (n = 152), (2) examination of the lawfulness of a tax assessment (n = 17), (3) representation of the taxpayer to the tax administration (n = 13), (4) business start-up consulting (n = 7), (5) consulting and/or preparation concerning a business plan (n = 1), (6) preparation of an annual net income statement (n = 8), and (7) customs law consulting (n = 1). Thus, we mainly addressed private households, as opposed to corporate employees and managers regarding tax-preparation services.

### 3.2 Measures

To test the hypotheses, we designed questionnaire items based on the propositions of Fishbein and Ajzen [16] concerning RAA [26]. Further, to take taxpayers’ perceptions of tax-preparation services, their own financial performances and perceived behavioral control into account, we considered the studies of Saleh and Athari [35] and Mawad et al. [36]. We used a 7-point Likert scale for each item (from 1 = “do not agree at all” to 7 = “completely agree”). Fishbein and Ajzen [16] stated that “It is important to realize that there is no single reasoned action questionnaire. Each study requires the construction of a suitable questionnaire.” Therefore, we adjusted the original RAA framework to suit the context of quality labels for tax-preparation services, creating items that cover relevant behavioral beliefs about signaling and decision-making. For normative beliefs, we used recommendations as signals of important reference groups, whereas control beliefs mainly pertained to the identification and assessment of quality labels in the context of tax-preparation services.

We included several control variables (i.e., the most important task for which individuals would utilize tax-preparation services, important quality labels for tax-preparation services that are known to individuals, and trust in quality labels). The complete questionnaire can be found in the Appendix.

### 3.3 Statistical tests

To test our research model, we used the partial least squares (PLS) approach for SEM. When research aims to identify key drivers, or explain or predict the target construction, the PLS approach is more appropriate compared to covariance-based SEM [37]. Multiple regression analysis, as an example of variance-based SEM, develops parameters that “maximize the explained variance of the dependent constructs” [37]. We utilized SmartPLS 4.0.9.6 software to estimate our weightings using a path method, and determined the significance of the path coefficients using bootstrapping procedures with 5000 samples [37]. Furthermore, we analyzed the *post hoc* data by considering propositions for multigroup analyses [38,39] for individuals who had consulted a tax-preparation service before versus those who had not. To test our model and *post hoc* analyses, we conducted several statistical tests (see Appendix). First, we followed the requirements of Hair et al. [40] and Hulland [41] for reflective measurement models to test the reflective variables “attitude,” “perceived norms,” “perceived behavioral control,” “intention,” and “trust in quality labels.” The values for indicator reliability were above 0.7. The average variance was higher than 0.5, whereas the values for discriminant validity using the heterotrait–monotrait (HTMT) ratio were below 0.9 [40,42]. Second, concerning formative measurement models, we tested the formative variables “behavioral beliefs,” “normative beliefs,” and “control beliefs.” For these, multicollinearity was deemed to have been avoided because the variance influence factors were below 5. We also examined the relative and absolute importance of all significant indicators. Accordingly, we sought to ascertain whether the bivariate correlations were higher between an indicator and the variable than between indicators, in order to check for heterogeneity [40,43]. We concluded that no suppressors or collinear indicators were present. Additionally, to ensure that our model did not contain unobserved heterogeneity, we performed finite mixture partial least squares (FIMIX-PLS) segmentation according to Sarstedt et al. [44]. We used three segments and ascertained that the normed entropy statistic for the segments surpassed the threshold of 0.5. Furthermore, because we estimated that the summed fit of Akaike's information criterion with factor 3, combined with consistent Akaike's information criteria, was the lowest for the complete dataset, we concluded that the dataset did not contain unobserved heterogeneity. We validated the quality of our structural model using several tests. First, the standardized root mean square residual was used to ascertain the approximate fit of our composite and common factor models (Henseler et al., 2014; Hair et al., 2019). We obtained values of 0.056 for the composite factor model and 0.073 for the common factor model for the overall sample. For those who had previously consulted a tax-preparation service, we determined a composite factor model of 0.072 and a common factor of 0.095, while those who had not, we ascertained values of 0.059 (composite) and 0.066. Additionally, to investigate the predictive power of our model by focusing on the early antecedents, we determined PLSpredict values according to Shmueli et al. [45] using 10 repetitions. Our PLS-SEM Q^2^ prediction values were positive, and our prediction errors were not distributed highly symmetrically. Thus, we checked whether our PLS-SEM mean absolute errors (MAE) were lower than those of the linear regression model (LM) ones. For the complete dataset and individuals who had not previously consulted a tax-preparation service, all PLS-MAEs were higher than the LM ones. Hence, we determined no predictive relevance of the early antecedents according to Shmueli et al. (2019). In contrast, we estimated low predictive power for early antecedents concerning individuals who had not previously consulted a tax-preparation service. Nonetheless, to estimate the predictive power of our late, and hence direct, antecedents, we used the cross-validated predictive ability test (CVPAT) according to Liengaard et al. [46]. To do so, we checked whether the average loss difference of PLS-SEM was significant versus the indicator average and the linear model. Hence, our CVPAT showed that the predictive power of our late antecedents was strong for the overall dataset and medium for individuals that had previously consulted a tax-preparation service before and for those who had not.

For the *post hoc* analyses, we divided the dataset into two groups: taxpayers who had previously consulted a tax-preparation service (n = 114) and taxpayers who had not (n = 85). To avoid misinterpretations because of small sample sizes, we considered propositions for statistical power analyses according to Kock and Hadaya [47]. They proposed that effect sizes, which Cohen [48] determined for behavioral science, must be adjusted based on sample size. According to Cohen [48], f^2^-values of 0.002, 0.15, and 0.35 indicate small, medium, and large effect sizes, respectively. By considering Kock and Hadaya [47] and our samples, we determined the effect sizes for previously consulted versus not previously consulted using G*Power 3.1.9.7 [49]. Thus, the threshold for small effect sizes was 0.003 for the complete dataset, 0.005 for previously consulted, and 0.007 for not previously consulted. Next, we performed permutation tests to report on significant differences between our groups, following the recommendations of Sarstedt et al. [38] and Hair et al. [39]. Compared with bootstrap multigroup analyses, permutation tests yield better results, even when asymmetrical sample sizes are used [50], which is why permutation tests are superior [39]. We performed our tests with 5000 permutations, as recommended by Hair et al. [39]. Furthermore, we reported tests for measurement models and structural models according to Hair et al. [40] for the *post hoc* analyses as well. Finally, we investigated whether our significant *post hoc* analyses were meaningful using the measurement invariance of composite models (MICOM) procedure according to Hair et al. [39]. For the MICOM procedure, three steps were necessary. First, we had to treat every measurement model for every group alike, which meant that (a) the number of indicators per measurement model was equal, and (b) the measurement models were treated equally concerning the parameters and application of the test algorithms. Second, we checked whether compositional invariance was present, because the original correlation had to be significantly above the 5 % threshold. Third, we checked the equality of the composite mean values and variances. When we determined significant variances in age and perceived behavioral control, the mean values for age were not significant. Therefore, we concluded that there was partial measurement invariance and consequently employed standardized path coefficients to report differences..

**Table 1:** The descriptives of the variables are shown in Table 1.

Variable	Mean value	Standard deviation
Behavioral beliefs	30.82	10.26
Normative beliefs	26.75	9.44
Control beliefs	22.85	11.17
Attitude	5.52	1.03
Perceived norms	4.60	1.27
Perceived behavioral control	5.91	1.00
Intention	5.04	1.30

Table 1 Descriptive results (n = 199).

**Table 2:** The correlations among the variables are presented in Table 2.

	Behavioral beliefs	Normative beliefs	Control beliefs	Attitude	Perceived norms	Perceived behavioral control	Intention
Behavioral beliefs	–	.607**	.771**	.783**	.589**	.511**	.797**
Normative beliefs		–	.646**	.543**	.721**	.341**	.520**
Control beliefs			–	.673**	.669**	.446**	.773**
Attitude				–	.562**	.481**	.736**
Perceived norms					–	.224**	.657**
Perceived behavioral control						–	.393**
Intention							–

Table 2 Correlations among variables (n = 199, **p < .01).

## 4 General results

### 4.1 Descriptives and correlations among variables

We found a high correlation between RAA variables (see Table 2). Although RAA components are conceptually different, high correlations are likely to occur [16]. Nonetheless, the HTMT in Section 3 demonstrated that the quality of the research model was not affected and was, thus, applicable to the scenario.

### 4.2 Detailed outcomes of the research model

**Fig. 2:**
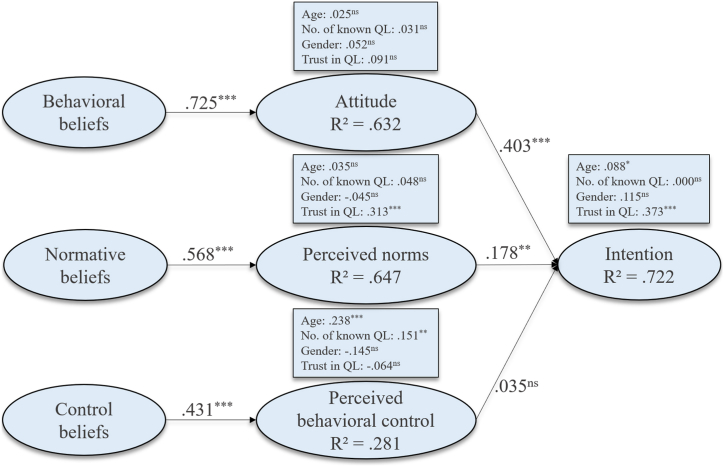
Research model results (Notes: *p < .05; **p < .01; ***p < .001; QL = quality labels; one-tailed tests).

The results of the research model are shown in Fig. 2.

### 4.3 Results regarding our hypotheses and control variables

According to the research model results shown in Fig. 2, we found strong empirical evidence for H1 (0.725; p < .001; f^2^ = 0.786), H3 (0.600; p < .001; f^2^ = 0.585) and H5 (0.431; p < .001; f^2^ = 0.141). Thus, H1, H3, and H5 are supported. According to the R^2^ value of an individual's intention to consider quality labels when selecting tax-preparation services, 72.2 % of the intention's variance was explained by attitude and perceived norms. According to Hair et al. [40], the intention to consider quality labels when identifying effective tax-preparation services is substantially explained by our model, since R^2^ = 72.2 %.

With regard to H2 (0.403; p < .001; f^2^ = 0.295), H4 (0.178; p < .01; f^2^ = 0.058), and H6 (0.035; p > .05; f^2^ = 0.003), we determined that H2 and H4 were supported, whereas H6 was not. Additionally, we used control variables to further verify our findings by investigating the individuals' age, gender, number of quality labels already known to them, and the respective individuals’ general trust in quality labels (see Appendix). The significances we determined were age toward perceived behavioral control (0.238; p < .001; f^2^ = 0.072), trust in quality labels (QL) toward perceived norms (0.313; p < .001; f^2^ = 0.186), and intention (0.373; p < .001; f^2^ = 0.260). Table 3 summarizes the outcomes regarding the hypotheses.

**Table 3:** Hypotheses results.

Hypotheses	Results β-, p-, f^2^-values	Significant?
H1	.725; p < .001; f^2^ = 0.786	Yes
H2	.403; p < .001; f^2^ = 0.295	Yes
H3	.600; p < .001; f^2^ = 0.585	Yes
H4	.178; p < .01; f^2^ = .058	Yes
H5	.431; p < .001; f^2^ = 0.141	Yes
H6	.035; p > .05; f^2^ = .003	No

### 4.4 Results regarding *post hoc* analyses

In addition to analyzing our hypotheses and control variables, we analyzed the *post hoc* data. Thus, we investigated (1) individuals who had previously consulted a tax-preparation service and (2) individuals who had not previously consulted a tax-preparation service. We determined a significant and meaningful difference in age regarding perceived behavioral control for individuals who had consulted a tax-preparation service before (0.345; p < .001; f^2^ = 0.164, see Appendix).

## 5 Discussion

### 5.1 Factors influencing the intention to consider quality labels for identifying effective tax-preparation services

In this study and to answer our research question, we determined a number of factors that influence the intention to consider quality labels when identifying effective tax-preparation services. First, we identified that attitude toward quality labels, that are based on behavioral beliefs, is the most important predictor of the intention to consider quality labels for identifying effective tax-preparation services. Hence, we support H1 and H2. This aligns with the insights of Witherspoon and Stone [3], who explained that tax-preparation services with quality labels receive better ratings on recommendation portals than do tax-preparation services without quality labels, which indicates a more positive attitude among users toward quality labels displayed by ratings. Second, perceived norms that are based on normative beliefs are relevant in determining the intention to consider quality labels when identifying effective tax-preparation services. Accordingly, we support H3 and H4. Hence, participants considered receiving recommendations from others regarding quality labels as important for identifying effective tax-preparation services. Thus, the results support the propositions of Sakurai and Braithwaite [9], who stated that family members, close friends, and colleagues are important groups to rely on when preparing income tax returns. Third, we determined that control beliefs are important to determine perceived behavioral control and thus we support H5 and propositions of Fishbein and Ajzen [16] that control beliefs are important. Unfortunately, we were not able to determine any influence of perceived behavioral control on intention, which is why we do not support H6. Fourth, we included several control variables (age, number of known quality labels, gender, and trust in quality labels). With respect to these control variables, trust in quality labels was an important predictor of the intention to consider quality labels when choosing effective tax-preparation services. Hence, participants rely on the fact that the information the quality labels provides about tax-preparation services is trustworthy. Thus, we support the propositions of Sakurai and Braithwaite [9], according to whom tax-preparation services must be trustworthy. This is because taxpayers have indicated that the honesty of tax-preparation services—that is, that they can be trusted to conduct legally compliant services—is the most important aspect when defining their ideal tax-preparation services. Further, Wheiler [4], Brown and Swartz [20], Silvestro et al. [5], Lee [11], and Luk and Layton [7] have revealed that, for professional service providers, and specifically tax-preparation services, measures are necessary to overcome expectation gaps for such services. Quality labels reduce such principal–agent dilemmas, such as expectation gaps concerning professional service providers [10]. In terms of gender influence regarding the intention to consider quality labels when choosing good tax-preparation services, the results contradict existing knowledge: While Fleischman and Stephenson [25] determined that women are more likely to use tax-preparation services than are men in order to stay legally compliant and save time, we were unable to determine significant differences in terms of gender among taxpayers.

### 5.2 Insights from *post hoc* analyses

Concerning *post hoc* analyses and in contrast to Stephenson et al. [26], we did not determine any statistical significances concerning the gender of our participants. Despite, we determined that age is positively related to perceived behavioral control for individuals who have previously consulted a tax-preparation service. Hence, the older the participants, the more confident they were in evaluating their self-efficiency in terms of their need to use quality labels when choosing a good tax-preparation service. Therefore, we support propositions of Dubin et al. [6] who state the age is important for choosing a good tax-preparation service. Thus, we propose that experiences in terms of life and former services are important for individuals to ascertain the use of quality labels to identify effective tax-preparation services.

### 5.3 Interpreting descriptives

Concerning descriptives (see Table 1), the mean values for attitude (5.52) and intention (5.04) show that quality labels are appropriate signals for individuals in facilitating decisions, and that these individuals use quality labels when choosing effective tax-preparation services. Accordingly, we recommend that tax-preparation services pursue the conferment of quality labels for their services as part of their marketing efforts. This is important for future tax-preparation services, as quality labels represent that the services are good, according to Stephenson et al. [26].

## 6 Conclusion

We analyzed the relevance of quality labels in identifying effective tax-preparation services from the taxpayer perspective. Our explained variance values indicate that intention to consider is well predicted and hence that relevant aspects in the context are covered. This confirms that RAA is an appropriate approach for determining the intention to consider quality labels when choosing effective tax-preparation services. Furthermore, the results show that individual attitudes and social norms of the relevant reference groups positively influence the intention to consider quality labels when identifying effective tax-preparation services.

### 6.1 Theoretical implications

This study's design and findings contribute to the accounting literature in several ways. First, by considering critical perspectives, we contribute to our understanding of moral hazards in accounting. We do so by addressing quality labels as an appropriate measure to reduce the principal–agent dilemma between taxpayers and tax-preparation services. Thus, we contribute to the professional service provider and principal–agent theory literatures by proposing that taxpayers and tax-preparation services use quality labels to overcome expectation gaps. The results help to explain when and why quality labels act as signals and are used to identify effective tax-preparation services, and we reveal the factors that determine the acceptance of quality labels. Second, we add to the knowledge of how forms of recommendation, specifically quality labels, in the tax-preparation service context are accepted by taxpayers. Although related work has focused on user ratings to determine the importance of quality labels in context [3], we provide insights concerning the intention to consider individuals. Third, we introduce RAA to analyze the intention to consider quality labels in a taxpaying context. The explained high variance confirms that the theory is helpful in understanding the reasons for consideration of intention. Thus, we contribute specifically to accounting and human behavior literature since, to the best of our knowledge, this study represents the first to examine psychological reasons for considering quality labels in identifying effective tax-preparation services. Fourth, the extension of RAA to cover trust contributes to the understanding of trust-related decision processes and evidence of trustworthiness—namely, quality labels. Thus, we propose that future studies seeking to identify the psychological reasons for considering signals in a service context should consider trust. Fifth, as age is significant for the intention to consider, we demonstrate that demographics matter and should be considered when analyzing the acceptance of quality labels in an accounting context.

### 6.2 Practical implications

From a practical perspective, we first recommend that tax-preparation services invest in being awarded quality labels, because the results show that quality labels are important for taxpayers in identifying effective tax-preparation services. Furthermore, the older the taxpayers become, and the more complex their tasks, the more important quality labels are in displaying special knowledge of tax-preparation services. Second, we recommend that institutions confer quality labels to good tax-preparation services so that tax-preparation services save money and time for taxpayers, ensure that tax services are legally compliant, and provide protection from the tax authority. This is because we determined that quality labels are important in assessing good tax-preparation services, and the aforementioned aspects are relevant motivators for taxpayers to utilize tax-preparation services [25]. Third, we propose that taxpayers who are seeking tax-preparation services should consider quality labels, because the results highlight the importance of quality labels and related work has revealed that tax-preparation services with quality labels perform better than do tax-preparation services without quality labels in terms of taxpayer ratings [3].

### 6.3 Limitations and future research

This study has several limitations that should be addressed in future research. First, since we used RAA as the theory for our research, and investigated the intention to consider quality labels when seeking tax preparation services but not the actual behavior, our study is subject to an intention–behavior gap [16]. Although we know the underlying reasons for the intention to consider quality labels when choosing effective tax-preparation services, there might be factors that were not examined in this study that would cause an individual not to consider quality labels. Thus, future studies should address the actual consideration behavior of taxpayers toward quality labels when identifying effective tax-preparation services, and the factors that lead to their failure to consider quality labels. Second, although taxpayers consider quality labels when identifying effective tax-preparation services, such labels might not ultimately be important in the selection process of preparation services. Hence, there may be other important factors, such as the price or availability of such services, that may lead to the selection of a specific tax-preparation service. Thus, future research should investigate the most important factors that lead to the selection of a tax-preparation service, and seek to determine the role that quality labels play in the decision-making process. Third, as three-quarters of the participants in our study stated that they would use tax-preparation services for their annual income tax returns, we assume that our participant group represents private tax interests, in contrast to company-related interests. Investigating company representatives could lead to different results and should be addressed in future studies to compare the results. Fourth, the participants of this study were selected from a single country, Germany. Future research should also consider other countries to identify additional relevant factors. Specifically, as Germany is one of the leading taxpaying OECD countriesKlicken oder tippen Sie hier, um Text einzugeben., studies should be conducted in countries with low or medium tax levies according to the OECD.

## Data availability statement

Repository: Open Science Framework.

DOI: 10.17605/OSF.IO/UE3KX.

## Additional information

No additional information is available for this paper.

## Ethics declarations

All participants provided informed consent to participate in the survey and to publish their data anonymously.

## CRediT authorship contribution statement

**Johannes Wichmann:** Writing – original draft, Project administration, Methodology, Formal analysis, Data curation, Conceptualization. **Michael Leyer:** Writing – review & editing, Validation, Supervision, Funding acquisition, Formal analysis, Data curation. **Isabell Altmüller:** Methodology, Formal analysis, Data curation, Conceptualization.

## Declaration of competing interest

The authors declare that they have no known competing financial interests or personal relationships that could have appeared to influence the work reported in this paper.

## Appendix

The data and analyses for our study are available here: OSF.IO/UE3KX.
